# Different Patterns of Mental Health Problems in Unaccompanied Refugee Minors (URM): A Sequential Mixed Method Study

**DOI:** 10.3389/fpsyt.2020.00324

**Published:** 2020-04-28

**Authors:** Bernd Hanewald, Michael Knipper, Werner Fleck, Jörn Pons-Kühnemann, Eric Hahn, Thi Minh Tam Ta, Burkhard Brosig, Bernd Gallhofer, Christoph Mulert, Markus Stingl

**Affiliations:** ^1^Centre for Psychiatry and Psychotherapy, Justus-Liebig-University Giessen, Giessen, Germany; ^2^Institute of History of Medicine, Culture, Migration & Global Health, Justus-Liebig-University Giessen, Giessen, Germany; ^3^General Practitioner, Giessen, Germany; ^4^Institute of Medical Informatics, Justus-Liebig-University Giessen, Giessen, Germany; ^5^Department of Psychiatry and Psychotherapy, Charité – Universitätsmedizin Berlin, Berlin, Germany; ^6^Department of Family Psychosomatics, Justus-Liebig-University Giessen, Giessen, Germany

**Keywords:** unaccompanied refugee minors, trauma, mental disorders, screening, Refugee Health Screener (RHS-15), children and adolescents

## Abstract

Unaccompanied refugee minors (URM) represent one of the most vulnerable refugee groups due to their young age, developmental status, and insufficient coping strategies. Clinical observations indicate that the frequency of mental health problems varies between different URM subgroups. In the present research project, clinical interviews as a source of qualitative data were combined with quantitative psychometric information in a mixed-method approach in order to study the patterns of mental health problems in 561 URM from four different language groups (Arabic, Farsi, Somali, and Tigrinya) immediately after arrival in the host country (Germany). Qualitative analysis obtained as differentiating categories “language, countries of origin, age, and gender”; quantitatively, the Refugee Health Screener (RHS-15) was applied. According to the positive screening results, the highest number of mental complaints was returned by children and adolescents speaking Farsi (65.9%) and Somali (65.8%). They were followed by URM speaking Arabic (49.4%) and Tigrinya (43.3%). The results were influenced not only by origin, but also by age (with higher burden among older Farsi-speaking URM) and gender (with higher burden among male URM). Although the prevalences in URM subgroups differ, the observed high rates of positive screening results in our sample of URM from Germany substantiate the need for early detection of mental complaints and appropriate mental health care for at least every second URM.

## Introduction

Unaccompanied refugee minors (URM) are distinctly different from other refugee groups because of their younger age, earlier psychosocial development stage, and deprivation from parental or any other adult's care. URM therefore constitute a vulnerable subgroup with a special need for protection. Accordingly, the reception directive of the European Parliament and the Council of Europe from 2013 obliges member states to take special account of particularly vulnerable refugees (1).

Like all minors, URM are more dependent on adult care at a younger age than close to adulthood. The lack of emotional, physical, financial, and emotional parental support both during (pre-)migration and the post-migration period has been demonstrated to significantly increase the risk of developing mental health problems in URM (2–6). Accordingly, Bean et al. (7) reported higher levels of traumatic stress symptoms in URM compared to adolescent refugees who had arrived with their parents. Before fleeing from their country of origin, URM often face social upheaval and chaos in their region. They experience threats regarding their own safety and/or the safety of a family member or a close person; they may witness or become engaged in violence, e.g. witnessing murders, mass killings, or having combat experience, affecting their moral perspectives (3, 8).

During migration, URM are frequently endangered, forced to survive often dubious transitional placements and face uncertainty about the future. The migration process can take much longer than expected, with different steps, interruptions, and times of detention. Adolescents from Afghanistan, for example, often stay in Iran or decide to move on to Europe at a later stage. Migrants from Somalia and Eritrea are frequently held hostage during their routes through Sudan and Libya, and families back home are forced to pay ransom. Many are killed and others suffer torture, violence, and sexual abuse. The final step on their way to Europe by boat also involves multiple threats and the risk of perishing or witnessing others drowning in the sea.

URM undergo these complex migration journeys at a young age amid important stages of psychological development and without the protection of adult minders. Such journeys will often take months or even years. Traumatic events during flight are likely to have devastating effects on URM's basic trust in the ability of adults to care for them. The latter include sexual exploitation and violence even after arrival in the host country (8–12). Yet the ability of children and adolescents to withstand external stress depends to a large extent on the emotional state of their minders. It has been reported that the separation of children from their parents may have a greater impact on the mental health of children than acts of war (13, 14).

The next stage of the migration process—arrival in the country of destination—may equally involve potentially stressful and harmful aspects.

Despite the experience/hope of having found a refuge in a safe place, the latter includes feelings of loss towards their homeland as well as distance from family and peers. In some cases, successful migrants even experience feelings of “survivor guilt” caused by the fact of being still alive while close persons were injured or killed (15). Frequently, the families left behind expect support with pursuing their own migration plans, financial aid by remittances or at least efforts towards reimbursement of money invested for smugglers and/or ransom. Added to this, the urge to cope with a new environment arises immediately upon being confronted with an unknown social and cultural world with a foreign language and, importantly, administrative demands regarding asylum procedures which are often difficult to fathom. The psychological impact of detention and human rights restrictions in the context of migration policies has been reported widely (12, 16–18), especially in the case of traumatized refugees.

The described complex of problems may place additional psychological strains on migrants (19–21), particularly on single children and adolescent URM without emotional protection, support, or security provided by family members (4, 22).

Concerning mental illness related to such psychosocial stressors, Jenssen et al. (3) reported that 54% of 93 URM in the care of the State Child Protection Services in Norway suffered clinically relevant posttraumatic stress symptoms, 30% showed anxiety symptoms, and 20% displayed symptoms of depression. Furthermore, the UMR in this study had experienced 5.5 stressful life events on average (e.g. death of a loved one, drastic changes in the family, separation from family against will, war or armed military conflict, experience of physical violence or witnessing of physical abuse), showing a significantly positive correlation with posttraumatic stress, anxiety symptoms as well as symptoms of depression. Vervliet et al. (23) assessed the mental health of 307 URM upon arrival in their host countries (Norway and Belgium). They found a high number of traumatic experiences in URM, e.g. death of a loved one, physical maltreatment and the experience of being in danger. Duncan (24) reported high rates of symptoms of posttraumatic stress disorder (PTSD) in 168 Sudanese refugee children in a Kenyan refugee camp, with almost 75% of them suffering from moderate to severe symptoms. In more detail, Derluyn et al. (25) mentioned that URM girls displayed more anxiety symptoms, emotional problems, and higher avoidance scores than boys, while boys had more difficulties with exhibiting prosocial behavior than girls. Moreover, sexual abuse was more frequent among unaccompanied minors compared with refugee minors with families. Within the group of URM, girls were especially prone to sexual abuse (26), Vervliet et al. (23) reported that in a cohort of 307 URM, upon arrival in Norway and Belgium high scores were found for anxiety (38 percentage points), depression (44 percentage points), and PTSD (53 percentage points). Regarding the severity of PTSD and depression symptoms, impairments in mental health were significantly associated with the accumulation of traumatic events and the lack of refugee status (3, 20). More specifically, four domains of traumatic events, namely abuse of human rights, lack of basic necessities, traumatic loss, and separation from others, were associated with symptom severity (27).

Taken together, recent studies have highlighted that the heightened risk of developing mental health problems in URM is due to multiple factors, such as high numbers and the extent of traumatic experiences in the country of origin and during flight, gender, and post-migration factors (9, 23, 28–30).

In 2016, 36,000 URM reached Germany seeking asylum (31). In Germany, upon arrival URM are distributed to different regions according to an administrative algorithm. Giessen, a small university town in the federal state of Hesse in central Germany, is host to one of the largest and most longstanding reception centers for refugees in Germany. Hence, the number of URM arriving here is particularly high.

The described high vulnerability and high rates of PTSD, depression, and anxiety disorders in URM identified in clinical trials were in line with our clinical observations within the first routine medical examination of the URM in Giessen. However, we also noticed recurrent variations of complaints within the different URM subgroups. In order to analyze these clinical observations and derive an overarching multidisciplinary explanatory approach based on clinical observation and enriched by these experiences, we applied a *mixed-method research design* (32, 33) to identify the differentiating factors influencing the mental health issues within the URM cohort during clinical routine. As an overarching goal we tried to implement a valid and economical procedure within the first medical examination. This was done related to the reception directive of the European Parliament and the Council of Europe form 2013, that obliges member states to take special account of particularly vulnerable refugees (European Parliament, European Council 2013). Against this obligation, in Germany up to now no standardized procedure has been established.

## Methods

Our research approach starting from clinical experiences corresponds to a mixed-method research design which combines elements of qualitative and quantitative research approaches (34). Specifically, we used an explanatory sequential mixed design: In the first step, we collected *qualitative* data by conducting narrative biographical interviews of UMR within the medical examinations. These semi-structured and problem-centred interviews (35, 36) with “anker-points” related to, among others, biography, family, education and reasons for flight took about 45–60 min.

Our strategy to analyze the qualitative data/interviews was a hermeneutic one. Aim of this procedure is to define supra-individual categories by clustering the narratives on common themes. This is conducted by a five-step approach: 1. Paraphrasing the contents of the narrative interviews. 2. Assigning the obtained paraphrases to themes/headlines according to a biographical interview guide. 3. Collecting interviews with similar content and unifying the headlines 4. Conceptualizing: similarities and diversity are formulated in terms of theoretical information and empirical knowledge. This step translates the original terminology into scientific words. 5. Theoretical generalization: relating the results to theories and supposed relations (37).

In doing so, we subsequently analyzed the obtained material and observed significant differences in the living conditions for UMR and the descriptions of mental health complaints across the different countries of origin. To systematize the findings, we formed inductively distinct categories.

In the second step, we used the RHS-15 as a *quantitative* measure to test the initial qualitative results of group differences in mental health problems. Target criteria of the Refugee Health Screener (RHS-15), developed by Hollifield et al. (38), are symptoms of depression, anxiety, PTSD, and physical concomitants as well as the subjective self-efficacy of the interviewed persons. The RHS-15 was derived from the most commonly mentioned complaints of refugees that have been documented in literature. To avoid potential harm triggered by answering the RHS-15, it doesn’t ask about any names or details of specific traumatic experiences. The questionnaire lists 14 Likert-scaled questions and an additional visual analog scale (“distress thermometer”). Based on a sum score >11 of the items 1–13 and/or a distress >5, the screening result is evaluated as “positive”. This evaluation strategy was derived from the results of the validation studies by Hollifield et al. (38). The overall procedure appears to be economical and easy to apply in large-scale screening of the mental health status of refugees (39). Cultural sensitivity and equivalence of the different language versions of RHS-15 have been shown (40). Within a German refugee sample, the different language versions of the RHS-15 showed excellent internal consistencies with Cronbach's α ranging from .91 to .93, and good convergent and predictive validity (41). The practicability and efficiency of the RHS-15 have been demonstrated in previous studies (39).

The total scores of the RHS-15 (RHS_total_) representing the severity of symptoms (items 1 to 13) were not distributed normally, as indicated by the Shapiro-Wilk-Test (for all items p < .001). Therefore, the data were quantitatively analyzed for significant group differences with nonparametric tests (Kruskal-Wallis-Test, Mann-Whitney-Test). In addition, Spearman correlations were applied to assess covariations with age. Given the skewed distribution of the data, we decided to report the medians and the 0.25 resp. 0.75 quartiles. All calculations were performed with IBM SPSS-Statistics 24 software.

In the final step, we integrated the two data spheres of qualitative and quantitative results and discussed them by adding objective country-specific information from public databases in order to provide an in-depth explanation for our findings.

### Study Cohorts

The URM were initially admitted to a central reception facility for refugees operated by the German state of Hesse and then eventually referred to the youth welfare office. Within the first week of their arrival, a general practitioner (GP) is assigned to conduct the initial medical examination and provide medical care in accordance with The Asylum Seekers Benefits Act and the Infection Protection Law. This physical examination was supplemented by medical history, dental status, blood tests, a stool sample, and chest X-ray. In order to additionally assess the mental status of the UMR during the initial examination, the responsible GPs extended the mandatory physical examination to include a psychological assessment and employed the RHS-15 screening supported by interpreters. The RHS-15 survey was voluntary. The resulting data helped to provide a more complete assessment of the young persons' health status and were made available to improve psychosocial care in shelters provided by the welfare organization responsible.

The total study cohort consisted of 561 URM from 4 different language groups (Arabic, Farsi, Somali, and Tigrinya) attending their first medical examination from 2015–2017. They represented various countries of origin: the majority of URM spoke Farsi (39.8%) and originated mainly from Afghanistan. They were followed by Tigrinya speakers from Eritrea (30.0%). Arabic speakers (16.3%), mainly from Syria, Iraq, Algeria, and Morocco, came next. The smallest language cohort were Somali speakers from Somalia (14.0%). The study sample was found to be representative with regional distribution of origin of the major groups of URM in Hesse in 2016 (42). The mean age of the children and adolescents was 17.7 years (SD=1.01), ranging from 14 to 19 years. 124 of them were female (23.7%) and 366 were male (76.3%). See **Table 1**.

**Table 1:** Sample characteristics relating to language resp. land of origin (Arabic = Syria; Farsi = Afghanistan; Somali = Somalia; Tigrinya = Eritrea).

Language	Arabic	Farsi	Somali	Tigrinya
Gender				
Male (n, %)	74 (87.1)	189 (90.9)	53 (72.6)	74 (47.1)
Female (n, %)	11 (12.9)	19 (9.1)	20 (27.4)	83 (52.9)
Age (mean, SD)	17.89 (1.07)	17.68 (1.17)	17.79 (0.91)	17.74 (0.80)

Subjects were excluded from further analysis if more than one item from the RHS-15 was missing (n=13), or if information about age or gender (n=25) was not available (total exclusion=6.77%). If only one item was missing, the missing value was imputed from the mean value of the specific item within the corresponding language group (6 item values). Overall, this left 523 URM for the final statistical analysis.

At the time of arrival in Germany, all participants were under the age of 18 years and had all migrated without being accompanied by any adult person older than 18 years of age. At the time of the study, some URM were already 18 or 19 years old due to the time interval between arrival in Germany and the onset of the study.

After receiving a positive decision from the ethics committee of the medical faculty of Justus-Liebig-University Giessen, the collected data were evaluated scientifically *post hoc*.

## Results

### Analysis of the Qualitative Data

As a result of the qualitative analysis, we identified relevant content differences determined by the supraordinate category “land of origin” representing the different impacts of long-lasting social and political conditions on vulnerability and resilience for mental health. The specific context factors relating to the global historical-political situation were evaluated by comparing the narratives with in-depth country-specific enquiries acquired from the UNICEF or UNHCR databases and related to the current literature in the field. To illustrate our conclusions, we subsequently present four illustrative biographical case reports of interviewed URMs with these origins.

As we conducted a post-hoc analysis, the case reports were taken from the records of the medical examinations. All personal information contained therein (e.g. names, exact places of action) was changed or edited out to exclude the identification of the participants.

In general, by means of the medical examinations, we obtained the impression that the Somali- and Farsi-speaking refugees might suffer most from mental health complaints. Somalia has had a long-term history of warfare and combat, especially in the southern regions. In the narratives of the Somali URM, the impact of the al-Shabaab militia as well as rivalries and blood feuds between clans were reported to have affected the lives of the parents and families of the URM before they had left the country. The parental generation of the URM had to live under unstable and traumatic living conditions for many years, accounting for an insufficient development of resilience factors within the next generation. As data presented by the UNICEF/UNESCO Institute for Statistics in 2015 demonstrated, in Somalia less than every fifth child will receive the opportunity to attend a primary school (43). The same holds true for Farsi-speaking URM. The greatest share of them in our cohort came from Afghanistan. Decades of warfare within the country have increased the risk for transgenerational traumatization (8, 44). Many of the Afghans who had fled to Iran were forced to live a socially marginalized life there. Despite speaking the same language (Dari, Farsi), Afghans living in Iran are discriminated against because of their specific dialect and are subsequently often segregated. Frequently, there are no opportunities for these children to regularly attend schools. In 2011, 47% of the 15 to 24-year-olds were able to read (45). Amongst URM in Iran, child labor is a common feature. In order to survive, segregated children and adolescents are forced to work in leather factories, in the construction industry and brickworks. In Germany, adult asylum seekers from Afghanistan have a fairly small chance of success in the asylum procedure and/or have to endure uncertainty until a final decision concerning their asylum application has been made.

#### URM Guuleed - Somalia

Guuleed is a 15-year-old male from Somalia. His father died during the war. Guuleed lived with his mother in a village outside Mogadishu. As the oldest of four siblings, he helped his family and relatives on the small family farm. G. attended the religious school Madrasa for one year. The family experienced the presence of the Al Shabaab militia as a constant threat, and there were repeated violent attacks on villagers. At the age of 14, Guuleed escaped from Somalia via Sudan and the Sahara. He spent three months in prison in Libya, and three days on a boat on the Mediterranean Sea prior to his arrival in Italy, with onward journeys to Switzerland and finally Germany. The clinical examination found persistent hepatitis B as well as an infection with Lambliasis. The RHS-15 was positive. In the care group, he withdraws, does not speak much, and preferred to play football all day long. He was transferred to a special youth welfare facility which takes care of adolescents suffering from traumatic disorders.

#### URM Ramin – Afghanistan

Ramin is a 14-year-old male from Afghanistan. He had to flee from Afghanistan with his mother and siblings at the age of three after the Taliban murdered his father. Ramin grew up in difficult conditions in Iran, did not attend school and received no health care. At the age of 14, he came to Germany without his family from Iran via Turkey and the Balkan route. Prior to his arrival in Germany, he was located in France. In Germany, he was taken into custody by the youth welfare office and lived in a youth welfare institution. Again and again, he displayed aggressive breakthroughs requiring psychiatric treatment. He had stomach problems and a plethora of functional complaints. He reported heavy mental burden in the RHS-15. Ramin learned to speak German quickly, obtained a high school diploma, and started vocational training. After a significant reduction in his medical drug intake, he was engaged as a youth representative for his firm, finished with a good final grades, and received support with behavioral therapy. To begin with, he had a scarcity of mental resources, but ultimately he was able to achieve a moderate level of stability with the help of external structures (school, training, medical support).

In contrast with a higher proportion of complaints among Somali and Afghan URM, children and adolescents from Eritrea and Syria seemed to report fewer mental complaints. In Eritrea, sporadic battles used to occur on the border with Ethiopia, but there was no civil war going on in the country. Regular schooling is commonly available, with a 93% literacy rate for citizens aged 15 to 24 reported in 2015 (45). Nevertheless, the government is autocratic and political tensions between government supporters and opposition groups as well as human rights violations have been widely reported. Attempts to escape compulsory military service are a frequent cause of migration and flight (46, 47).

Most of the Arabic-speaking URM are refugees from Syria. In Syria, mandatory education is provided for nine years. The literacy rate for adolescents over the age of 15 in 2013 was approximately 96% (45). Prior to the onset of the civil war in 2011, the country had low crime rates and comparatively stable living conditions.

URM from Eritrea and Syria were thus only temporarily exposed to distressing events and conditions of threat. Before the onset of the civil war, young people in Syria had the chance to grow up in a comparatively stable society, with a well-developed health and education system. The social environment offered opportunities to develop resources and resilience within a—relatively to Afghanistan and Somalia—quite stable framework, with positive features of education, social support, and stable family structures. In addition, refugees from Syria and Eritrea have better prospects for remaining in Germany, unlike refugees from Afghanistan and Somalia. The expected chances of success in the asylum procedure after reaching adulthood also seem to have an impact on the mental health of URM.

#### URM Kidane - Eritrea

Kidane is a 15-year-old- male from Eritrea. His father is in prison; an older sister lives in Ethiopia. Kidane lived with relatives in Asmara. He liked to play football and ride a bike and attended school in Eritrea for 7 years. There was an increasing threat of being conscripted for military service and he was afraid because his father is in prison. After fleeing the county, he spent 2 years on the way from Eritrea to Germany without any contact with his parents during this time. He suffered a car accident while in the Sahara. Later, Kidane spent 5 months in prison in Libya, where he was frequently beaten and his right collar bone was broken. He underwent surgery in Switzerland. At the clinical examination in Germany he was found to be underweight. Scabies scars and amoebae in his stool were found. The RHS-15 was negative. Kidane integrated well into the care group and was transferred to a youth welfare facility. He wanted to continue to attend school and get an education. He wants to support his relatives in Eritrea financially as soon as possible.

#### URM Elvedin - Syria

Elvedin is a 16-year-old male from Syria. In his home country, Elevedin went to school for 8 years. During the civil war, he witnessed explosions and attacks with helicopters and planes. He left his home with his family and lived in a camp in Syria. Then, Kidane left Syria with his uncle, but without his parents. Subsequently, he lost his uncle in Turkey. Kidane had only sporadic contact with his parents via cell phone and was very worried. It took him 6 weeks to travel from Syria to Germany. In Austria he was imprisoned for one week. Finally, an attempt to cross the border to Germany was successful. No physical abnormalities were found and the RHS-15 was negative. He hopes to learn German and attended school as quickly as possible. Kidane intends to study computer science, his greatest wish is to live with his parents in Germany in the future.

These short case vignettes exemplify the domains we identified as distinct factors contributing to the mental health status of URM within the narrative interviews. Firstly, we found similarities between the narratives of the Somali-Afghanistan cohorts and the Eritrean-Syrian cohorts of URM. The analysis of these patterns resulted in the constitution of different categories to explain the observed differences in mental health of the URM subgroups. As mentioned above, this framework is based on political-historical and psycho-social factors determining the living conditions of the URM in their homeland and makes it possible to identify and contrast the facets of URM's individual experiences and their impact on child development. In addition to the category “land of origin”, the qualitative analysis of the narratives revealed that the factors “age” and “sex” further contributed to the variance in the URM's mental health. This is in line with the empirical findings described in the sections above.

### Analysis of the Quantitative Data: Refugee Health Screener (RHS-15)

In order to quantify the qualitatively determined different patterns in mental health complaints, we ascertained the extent of mental health complaints in the study sample using the RHS-15 and analyzed the results related to the category factors “land of origin, age and sex”.

**Figure 1:**
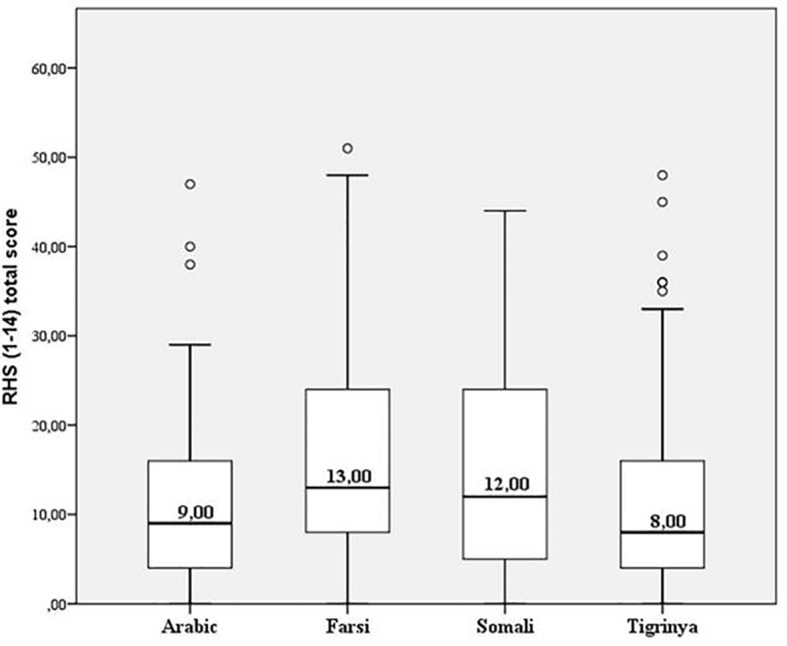
Distribution of symptom load (Median of RHS total scores) relating to language resp. country of origin (Arabic = Syria; Farsi = Afghanistan; Somali = Somalia; Tigrinya = Eritrea). The boxes represent the ranges from the 0.25-quantile to the 0.75-quantile.

The internal consistency of the RHS-15 was high with a Cronbach's α=.895. As a global outcome, we found that 43.6% of the study participants were rated as “positive” in the RHS-15. This implies that this share is highly likely to be suffering from at least one mental disorder (**Figure 1**).

With regard to the different languages/versions of the RHS-15, URM speaking Farsi (65.9%) and Somali (65.8%) showed the most frequently positive screening results, followed by Arabic (49.4%) and Tigrinya (43.3%) speakers. Upon differentiating the number of symptoms (RHS_total_) with Kruskal-Wallis-Tests, we found a significant difference between the language cohorts (n=523; H(3)=31,528; p < .001). Pair-wise comparisons revealed significant differences between Tigrinya-Somali (H=58,674; p=.036), Tigrinya-Farsi (H=82.574; p=.000) and Farsi-Arabic (H=71.104; p=.002) (cf. **Figure 1**)

A significant association between age and degree of mental impairment was only found in Farsi-speaking URM (Spearman correlation: r =.152, p =.028) with older URM expressing more mental complaints than younger URM.

In general, male URM (n = 399; RHS_total_: Median= 10, 0.25 quantile (RHS-total)= 4, 0.75 quantile (RHS-total)=20; positive RHS-15 = 58.9%) were found to carry a higher burden of symptoms and more positive screening results compared to female URM (n = 124; Median= 5.5, 0.25 quantile (RHS-total) = 2, 0.75 quantile (RHS-total)=16; positive RHS-15 = 48.4%; Mann-Whitney-Test U=29995.00; p=.000).

## Discussion

URM are particularly at risk of mental health problems (9, 23, 28–30). During the first medical examinations of UMR, we observed qualitative differences between sample subgroups, mainly corresponding to land of origin, sex, and age. To address these differences, a valid screening procedure assessing mental impairment and trauma-associated symptoms of URM was applied and implemented within the routine (first line) examination by a GP. The RHS-15 was chosen because of its high sensitivity and simple mode of application (38). Without, however, specifying this in detail, the implementation of the RHS-15 in this context has proven its feasibility analogous to a prior study and is therefore recommended for wider use (39).

As an alarming result, almost half of the 523 URM included in the study (46.9%) returned a positive screening result, indicating that positively screened URM may suffer from at least one of the most common mental disorders, which are mainly expected to be PTSD, depression, and anxiety. The proportion of study participants rated as “positive” is comparable to the observations of Jakobsen et al. (48) and Derluyn & Broekaert (49). Jakobsen et al. (48) reported that 41.9% of unaccompanied asylum-seeking adolescents in Norway, mainly originating from Afghanistan and Somalia, had symptoms suggestive of at least one psychiatric disorder, mostly PTSD, major depressive disorder, agoraphobia, or general anxiety disorder. In the same way, Derluyn & Broekaert (49) highlighted that 37 to 47% of URM exhibited symptoms of PTSD, depressive disorder, or anxiety. Not least, in comparison to the general population, the observed substantially higher rates of indications for psychiatric disorders and mental health problems in refugee minors is in line with the results of a recently systematic review by Kien et al. (50), who found up to a third suffering from depression or anxiety and up to half being affected by PTSD. The authors of the review also stated a high heterogeneity of point prevalences, which may be influenced by the different traumatizing experiences in the home country or during migration and diverse challenges or problems in the host country.

### Differences Between Language Cohorts

In our study sample, significantly different patterns of mental health in URM were found depending on the language cohort: Somali and Farsi speakers reported much more mental strain and had higher positive screening results in the RHS-15 compared to Arabic and Tigrinya speakers. This finding points to the influence of origin on the extent of mental distress reported by URM upon arrival in Germany. In addition to the individual traumatic experiences, war-related reduced, or missed schooling specific to the different countries of origin, interruption of schooling during flight followed by higher demands due to lower language skills in asylum countries all contributed to huge levels of further migration stress (51, 52). Furthermore, as children's social adjustment and self-worth are mainly predicted by the quality of peer relationships, positive experiences in school, and leisure time were shown to be sufficiently protective factors in overcoming and processing of migration trauma.

### Influence of Age and Gender

A positive correlation between age and symptoms has already been reported by Bean et al. (53). Our study supports this finding for Farsi-speaking minors. One possible explanation for the age-effect could be that older minors may have experienced a higher number of traumatic life events. Another possible reason for the increase in mental symptoms with age in Germany could be the fear of deportation from Germany back to the respective countries of origin once the age of maturity is reached. Unlike European passport holders, among refugees, reaching the age of eighteen is not associated with more liberties, but rather with more fears. At present, deportations of URM before that age are prohibited in Germany. Repatriation is only permitted if family members, other legally entitled persons or a suitable host institution in the country of origin can provide proof of a safe and responsible environment. In a host of cases, reaching adulthood will also mean the end of youth welfare support. Former URM may be moved to a community shelter for asylum seekers of mixed backgrounds and legal custody will also end. In the worst case, reaching adulthood could mean mandatory deportation without legal protection.

Data on gender differences have provided controversial results. Sourander (6) found no gender differences in the frequency of psychiatric symptoms in URM. Seglem et al. (54) reported more depressive symptoms in female than in male URM, albeit the effect size was low. The meta-analysis by Huemer et al. (28) emphasized that female gender increases vulnerability for the onset of mental health problems in URM. In the latter study, female URM appear to be more prone to be victims of sexual violence. In our study, a higher symptom burden and more positive screening results were found in male adolescents than female adolescents. A possible explanation for the diversity of gender effects found in different studies may be the heterogeneity of the studied samples. The relation between the frequency of stressful life events and the occurrence of mental disorders could be an essential factor. Some studies reported an overall higher incidence of negative or traumatizing life events (e.g. familial changes, death of a loved one, war or armed conflict, physical maltreatment, sexual violation) in male URM than in female URM. In addition, higher PTSD scores in male URM than in female URM were reported in some of the studies (9, 10, 23, 53). Another reason may have been that the type and frequency of traumatic experiences were not systematically recorded in our study because there was concern that these factors could have re-traumatized the URM during the RHS-15 screening. Until further standardized studies concerning gender differences are available, the interpretation of data in this field will have to be interpreted in a prudent manner.

## Limitations

The RHS-15 is a screening tool for easy and widespread use. The screening is to be understood as a first measure to detect mental suffering in UMR and cannot replace a valid diagnostic by a child/adolescent psychiatrist. Implementing a screening procedure within the first medical examination should therefore be understood as a feasible first step embedded in a broader concept for support. Although it covers the most common mental health complaints on a symptomatic level, it cannot replace elaborated diagnostic classification. Although the RHS-15 has proven good predictive values for detecting the relevant symptoms (41), another aspect to be kept in mind is a possible overestimation of symptom frequency by using a questionnaire-based self-report-screener, which sometimes is extreme. For instance, Engelhardt et al. (55) showed that PTSD rates in veterans from clinical diagnostic interview were 41% lower than estimates obtained by self-report questionnaire. A positive screening result will, however, increase awareness of mental problems and therefore increase the chance that more essential steps will be undertaken as a consequence.

As a measure of caution, it must be pointed out that more specific psychopathological symptoms—such as psychosis and effects of a poor physical constitution—are beyond the scope of the RHS-15 and should be derived from the general practitioner's findings.

The representatives of obtained group differences are limited due to the RHS-15 assessment in the four language versions—although about 77% of the Afghans speak Farsi—the results cannot be generalized to all URM stemming from Afghanistan. The type and frequency of traumatic experiences should be systematically recorded in further studies to provide a better understanding of the obtained group differences.

Another potential bias could be the stage and long-term perspective of the individual asylum procedures. An earlier study conducted by our group demonstrated that the results of the RHS-15 were different between refugee groups immediately after arrival compared to those with a longer duration of residence in the country (39). Moreover, in the present study primarily factors that increased vulnerability were examined. URM were examined shortly after arriving in Germany and of course the migration process was not yet completed this point. Post-migration factors fostering resilience were not the subject of the study, although they can counterbalance vulnerability factors (56). Favorable post-migration factors such as social and professional support, education, religion, acculturation strategies, and hope may contribute to the fact that refugees can be both vulnerable and resilient at the same time and therefore can adapt well despite unfavorable starting conditions (57, 58). This duality should be given more attention in further studies.

## Conclusion

We performed an analysis of the current mental health condition in URM and related the findings to factors we extracted from the obtained interviews. In this last section we focus on the combined results of the qualitative and quantitative data.

Different non-trauma-related factors determine the risk of developing trauma disorders: origin, age, and gender can represent risk factors for the occurrence of post-traumatic disorders in URM. In particular, the situation in the country of origin before the occurrence of individual stress from trauma or flight seems to influence the subsequent coping options after flight. Longer substantial instabilities in the country of origin, associated with social stress factors and less school education, seem to influence individual resilience.

The alarming prevalences of mental health complaints identified substantiate the particular vulnerability of UMR. They highlight the need for adequate child and youth psychotherapy because it is known that untreated mental health problems such as PTSD might lead to chronic courses of disease (59) and severe behavioral problems (7). As other studies have previously underlined, early symptom detection is of critical importance in order to provide timely psychosocial intervention among URM in the form of a stepped care approach (60), comprising e.g. physical safety, adequate residential settings, and educational opportunities.

In our procedure, positive screening results during the first medical examination were therefore directly reported to the youth welfare office, paving the way suitable care and housing to be allocated. In some cases, however, the URM were relocated to other federal states after their initial medical examination and it remained unclear to what extent the need for special protection was considered in the further course of their cases.

Although capacities for psychiatric and psychotherapeutic treatment are still lacking, the attestation of a status of particular vulnerability could help to initialize preliminary stabilizing measures or psychoeducational elements. The provision of early treatment helps to prevent aggravation and continuation of emotional problems (61).

Suffering from mental disorders at a vulnerable stage of life such as adolescence can give rise to severe development difficulties, resulting in obstacles hindering social integration, problems of bonding capacity and competency in relationships as well as chronic courses of disease. Our results indicate that in addition to age and gender, the land of origin representing different historical-political and social developmental conditions moderates the extent of mental complaints and must be borne in mind when assessing URM.

Still, there is a lot of work to be done. The found factors influencing mental health of URM are not the only ones that are of crucial importance for URM´s health in the long run. Beside the conditions in the country of origin, the demanding act of migration itself, social and legal conditions, ethnic affiliation, discrimination in the host country, social support, and possibilities to have access to health care institutions are momentous for further health development (62). Further studies should focus similarly on pre-departure factors, occurrences on the escape-route as well as on post migration stressors. Lifespan perspective and prospective studies can enable a better understanding of the effectiveness of coping strategies and resilience factors (57).

It is crucial to manage the challenge of integration in order to prevent larger societal problems, as less integration has the potential to increase the risk of developing a mental illness. Therefore, early detection of URM at risk for developing mental impairments is an essential preventive measure to avoid later undesirable development in connection with higher costs and poorer integration. On the other hand, predominantly post-flight stressors on children and adolescents might favor negative outcomes and denying URM´s needs can lead to further societal and economic costs along with a lack of integrations actions (57).

The implementation of a screening procedure can therefore be considered “profitable” from an economic point of view. Health care costs, procedural difficulties and duration of proceedings can be reduced, while non- or inappropriate treatment might lead to a chronic course of stress reactions or even suicidal ideation (5).

In view of this, the early detection of mental health problems after arrival is especially important for both the URM and the host county. It enables adequate support during the vulnerable transition period from adolescence to adulthood as well as a successful transition to an integrated life in a new culture and society (60).

## Data Availability Statement

The datasets generated for this study are available on request to the corresponding authors.

## Ethics Statement

The studies involving human participants were reviewed and approved by the ethics committee of the medical faculty of Justus-Liebig-University Giessen. Written informed consent from the participants' legal guardian/next of kin was not required to participate in this study in accordance with the national legislation and the institutional requirements.

## Author Contributions

BH: Principal investigator and main author. MK: Transcultural expertise. WF: Data collection. JP-K: Statistical processing of the data. EH: Supporting the study design and the interpretation of the results. TT: Supporting the study design and the interpretation of the results. BB: Statistical and methodological support. BG: study design and editing the manuscript. CM: study design and editing the manuscript. MS: Principal investigator and main author.

## Conflict of Interest

The authors declare that the research was conducted in the absence of any commercial or financial relationships that could be construed as a potential conflict of interest.
